# DEPDC1 up‐regulates RAS expression to inhibit autophagy in lung adenocarcinoma cells

**DOI:** 10.1111/jcmm.15947

**Published:** 2020-10-05

**Authors:** Wei Wang, Aili Li, Xiaodan Han, Qingqing Wang, Jinyong Guo, Youru Wu, Chen Wang, Guojin Huang

**Affiliations:** ^1^ Laboratory of Respiratory Diseases the Affiliated Hospital of Guilin Medical University Guilin China; ^2^ Guangxi Key Laboratory of Molecular Medicine in Liver Injury and Repair Guilin Medical University Guilin China

**Keywords:** A549 cells, autophagy, DEPDC1, H1993 cells, HCC827 cells, lung adenocarcinoma, RAS

## Abstract

DEP domain containing 1(DEPDC1) is involved in the tumorigenesis of a variety of cancers. But its role in tumorigenesis of lung adenocarcinoma (LUAD) is not fully understood. Here, we investigated the role and the underlying mechanisms of DEPDC1 in the development of LUAD. The expression and prognostic values of DEPDC1 in LUAD were analysed by using the data from public databases. Gene enrichment in TCGA LUAD was analysed using GSEA software with the pre‐defined gene sets. Cell proliferation, migration and invasion of A549 cells were examined with colony formation, Transwell and wound healing assays. The function of DEPDC1 in autophagy and RAS‐ERK1/2 signalling was determined with Western blot assay upon DEPDC1 knockdown and/or overexpression in A549, HCC827 and H1993 cells. The results demonstrated that DEPDC1 expression was up‐regulated in LUAD tissues, and its high expression was correlated with unfavourable prognosis. The data also showed that DEPDC1 knockdown impaired proliferation, migration and invasion of A549 cells. Most notably, the results showed that DEPDC1 up‐regulated RAS expression and thus enhanced ERK1/2 activity, through which DEPDC1 could inhibit autophagy. In conclusion, our study revealed that DEPDC1 is up‐regulated in LUAD tissues and plays an oncogenic role in LUAD, and that DEPDC1 inhibits autophagy through the RAS‐ERK1/2 signalling in A549, HCC827 and H1993 cells.

## 1 INTRODUCTION

Lung cancer is one of the most severe malignancies with great morbidity and mortality in the United States and worldwide.^1^, ^2^ Cancer statistics have revealed that, among all cancers, lung cancer alone is the number one human killer and surpasses the next three most common cancer (colon, breast and prostate) combined.^3^ According to the histological features, lung cancer is divided into two subtypes: small cell lung cancer (SCLC) and non‐small cell lung cancer (NSCLC).^4^ SCLC accounts for about 15% of the total cases, and NSCLC accounts for about 85% of the total cases. NSCLC is further divided into three main histological subtypes: lung adenocarcinoma (LUAD), lung squamous cell carcinoma (LUSC) and large cell carcinoma.^4^ Significant progress has been made in NSCLC treatment during last two decades. Targeted therapy and immunotherapy, combined with traditional surgery, radiotherapy and chemotherapy, have made great contributions to improved survival of patients.^5^ However, due to the limitation of diagnosis, most NSCLC patients were already in the late stage,^3^ and thus, the 5‐year survival rate of patients was about low.^3^ To achieve better outcomes in NSCLC treatment, it is urgent to discover novel biomarkers, therapeutic targets, as well as drugs for NSCLC.

DEP domain containing 1 (DEPDC1) is an oncogenic molecule, which plays an important role in various malignant tumours, including bladder cancer,^6^ hepatocellular carcinomas (HCC),^7^ prostate cancer^8^ and breast cancer,^9^ and may serve as prognostic predictor in HCC^7^ and breast cancer.^10^ Katagiri and colleagues firstly reported DEPDC1 was abnormally up‐regulated in bladder tumours, but was not detectable in 24 normal human tissues other than testis.^6^ But the role of DEPDC1 in normal tissue is unknown. Harada et al further revealed that DEPDC1 may interact with zinc finger protein (ZNF224) to form functional complexes, thereby inhibiting the transcription of A20, leading to the translocation of NF‐κB from cytoplasm to nucleus, where the transcription of a set of anti‐apoptotic genes is activated.^11^ A peptide derived from DEPDC1 611‐628 amino acid residues (named as 11R‐DEP:611‐628) can disrupt DEPDC1/ZNF224 complex and thus may inhibit cell proliferation of bladder cancer.^11^ Our group found the 11R‐DEP:611‐628 peptide inhibited proliferation of lung cancer and liver cancer cells by inducing apoptosis.^12^, ^13^ There are several reports investigated DEPDC1's role in tumorigenesis of other cancers. Feng et al found that DEPDC1 was involved in cell cycle progression of nasopharyngeal carcinoma.^14^ Huang et al revealed that DEPDC1 interacted with E2F1 in prostate cancer cells and thereby increased the E2F1 transcription activity to activate the expression of the downstream genes, which promote cell proliferation.^8^ Guo et al showed that, in hepatocellular carcinoma cells, DEPDC1 might up‐regulate the expression of chemokine ligand 20 (CCL20) and chemokine receptor 6 (CCR6), and thus could drive cell proliferation and invasion through CCL20/CCR6 signalling axis.^15^ Zhao et al demonstrated that, in breast cancer cells, DEPDC1 could activate PI3K/AKT/mTOR signalling.^10^ Zhang et al showed that DEPDC1 could up‐regulate Forkhead Box M1 (FoxM1) expression to facilitate cell proliferation in breast cancer cells.^9^ How DEPDC1 is up‐regulated in tumours is unknown yet, but two studies reported respectively that DEPDC1 expression is negatively regulated by miRNA 130a and miRNA 26b.^9^, ^16^

Autophagy is an evolutionarily conserved pathway by which dysfunctional organelles and damaged proteins are captured and degraded into ingredients to be reused.^17^ Autophagy plays an important role not only in homeostasis, but also in diverse disease pathologies, including cancer, neurodegeneration and infections.^17^ Autophagy may act as tumour suppressor in tumour initiation period of tumourigenesis, but as tumour promoter in established tumours.^18^ Whether there is connection between DEPDC1 and autophagy in cancer cells remains unknown.

Our previous study revealed that DEPDC1 is expressed in multiple lung cancer cell lines and 11R‐DEP:611‐628 can induce apoptosis of LUAD A549 cells, indicating DEPDC1 plays its role by forming complex with ZNF224 in A549 cells.^12^ However, whether DEPDC1 impacts other signalling pathway(s) besides NF‐κB pathway is unclear. In addition, DEPDC1 expression in LUAD tissues and its potential prognostic value remain unknown. In the present study, we analysed DEPDC1 expression in LUAD tissues and its prognostic value, and investigated connection between DEPDC1 and autophagy and the underlying mechanisms.

## 2 MATERIALS AND METHODS

### 2.1 Cell culture and transfection

Lung adenocarcinoma cell lines A549, HCC827 and H1993 were obtained from the Kunming cell bank of the Chinese Academy of Sciences (Kunming, Yunnan, China). The cells were cultured in RPMI‐1640 medium containing 10% foetal bovine serum (Thermo Fisher Scientific, Waltham, MA, USA) and 1% penicillin/streptomycin at 37°C in a humidified incubator with 5% CO_2_. The cells (1.5 × 10^5^) were seeded into 6‐well plates and cultured overnight, and transfection was performed with the Lipofectamine 3000 (Invitrogen, Carlsbad, CA, USA) according to the manufacturer's instructions. 2 µg plasmids/well was used for overexpression experiments. DEPDC1 siRNA (si‐DEPDC1 hereafter, 75 pmol) and control siRNA (si‐control hereafter, 75 pmol) per well were used for RNA interference experiments.

DEPDC1 expression plasmids pCAGGSc‐DEPDC1‐V1‐HA and vector plasmid pCAGGScHA were kindly provided by Dr T. Katagiri (The University of Tokushima, Japan).^11^ The si‐DEPDC1 and si‐control were synthesized by GenePharma (Shanghai, China). si‐DEPDC1 sequence: #1, 5′‐CCCUAGAAGAAGUCAUAAATT‐3′; #2, 5′‐GGCCAAUACAAGUAAACGUTT‐3′; #3, 5′‐CGAGGUCACUGAUGAUACATT‐3′; si‐control sequence: 5′‐UUCUCCGAACGUGUCACGUTT‐3′.

### 2.2 Western blot analysis and antibodies

Western blot assays were performed as previously described.^13^ In brief, the cells were collected 48 hours after transfection and lysed with lysis buffer (cat. no. R0020; Beijing, Solarbio Science and Technology Co., Ltd.). The protein concentration of the supernatant was measured with a BCA kit (Beyotime Institute of Biotechnology, Shanghai, China) according to the manufacturer's instructions. 30 µg of protein of each sample was separated by 10% sodium dodecyl sulphate and polyacrylamide gel electrophoresis, and then transferred onto polyvinylidene difluoride membrane, which was blocked with 5% non‐fat milk at room temperature for 1 hour, followed by incubation with the primary antibody (LC3B dilution 1:1000, p62/SQSTM1 dilution 1:1000, β‐actin dilution 1:2000) at 4°C overnight. The membrane was then incubated with a horseradish peroxidase‐conjugated goat anti‐rabbit/mouse secondary antibody (dilution: 1:1000) at room temperature for 1 hour. Blots were developed using an ECL kit (cat. no. P0018, Beyotime Biotechnology). The density value of the target protein and β‐actin was analysed using ImageJ software (version 1.53a; National Institutes of Health, Bethesda, MD, USA). The primary antibodies used were as follows: anti‐DEPDC1 (cat. no. ap5428a‐400) was purchased from ABGENT. LC3B antibody (cat. no. nb600‐1384) and p62/SQSTM1 antibody (cat. no. nbp1‐48320) were purchased from Novus Biologicals LLC. Anti‐ERK and anti‐p‐ERK were from Abcam Inc (Cambridge, MA, USA). Anti‐S6K1, anti‐p‐S6K1, AKT and p‐AKT were from Cell Signaling Technology, Inc (Danvers, MA, USA). Pan RAS were from Proteintech Inc (Wuhan, Hubei, China). Anti‐GAPDH and anti‐actin were from ZSGB‐BIO (Beijing, China).

### 2.3 Colony formation assay

600 cells were seeded into each well of 6‐well plate, and then, 2 mL culture medium was added into each well. Cells were cultured at 37°C in a humidified incubator with 5% CO_2_ for 10‐15 days. Cells was washed with PBS 3 times, fixed with 4% neutral paraformaldehyde solution for 30 minutes and followed with 3 times PBS wash; then, 2 mL 1% crystal violet solution was added to each well and incubated at room temperature for 2 hours. The crystal violet solution was discarded, and cells were washed with PBS 3 times. After the plates were dry, the colonies were counted and pictures were taken to record.

### 2.4 Wound healing and transwell assays

For wound healing assay, the cells were cultured to full confluence in 6‐well plates. The cells were scratched using a 10‐µL pipette tip in the centre of the well. The cells were washed with PBS 3 times, then incubated in medium containing 2% FBS. Representative images were captured at indicated time after injury. The width of wound healing was quantified and compared with baseline values.

For transwell assay, 3 × 10^4^ cells in serum‐free medium were seeded into upper chamber without (migration) or with (invasion) Matrigel (BD Bioscience, San Jose, CA, USA). 1 mL RPMI‐1640 medium containing 20% FBS was added into lower chamber. After incubating for 24 hours, the upper chambers were washed with PBS 3 times, and then fixed with 4% paraformaldehyde for 10 minutes, further stained with 0.5% crystal violet for overnight and followed with PBS wash. The reverse sides of the upper chambers were photographed. Five fields were randomly selected to calculate cells that migrated or invaded.

### 2.5 LUAD datasets collection, DEPDC1 expression in LUAD and prognosis analysis

LUAD gene expression datasets GSE75037 and GSE31210 were downloaded from the Gene Expression Omnibus (GEO) public database, and from which DEPDC1 expression data in LUAD and normal tissues were retrieved and analysed using GraphPad Prism 5 (GraphPad Software Inc, San Diego, CA, USA). The DEPDC1 expression in TCGA LUAD and normal tissues was analysed using online software GEPIA (http://gepia.cancer-pku.cn).^19^ The prognostic values of DEPDC1 in LUAD were analysed using online software Kaplan‐Meier Plotter (http://kmplot.com).^20^ Log rank test was used for survival probability difference.

### 2.6 Gene set enrichment analysis

TCGA LUAD gene expression dataset was downloaded through Xena browser (https://xenabrowser.net/datapages). Tumour samples in TCGA LUAD dataset were classified into high‐ and low‐DEPDC1 groups using median value of DEPDC1 expression as cut‐off. Then gene set enrichment was analysed using GSEA 4.0.3 software (downloaded from http://www.broad.mit.edu/gsea/) with the pre‐defined gene sets: hallmark gene sets, oncogenic gene sets and curated gene sets. Permutation number was set as 1000. A gene set is considered significantly enriched when the false discovery rate (FDR) score < 0.25.

### 2.7 Statistical analysis

Statistical analysis was conducted using SPSS Statistics 20 (IBM, Armonk, NY, USA) and GraphPad Prism 5 (GraphPad Software Inc). All values were expressed as mean ± standard deviation (SD). All experiments were carried out independently at least 3 times. Comparison between two groups was performed using Student's *t* test, and comparison of multiple groups was conducted using one‐way analysis of variance (ANOVA) test followed by the Student‐Newman‐Keuls post hoc test. *P* < 0.05 was considered as statistically significant.

## 3 RESULTS

### 3.1 DEPDC1 expression is up‐regulated in LUAD tissues

**FIGURE 1:**
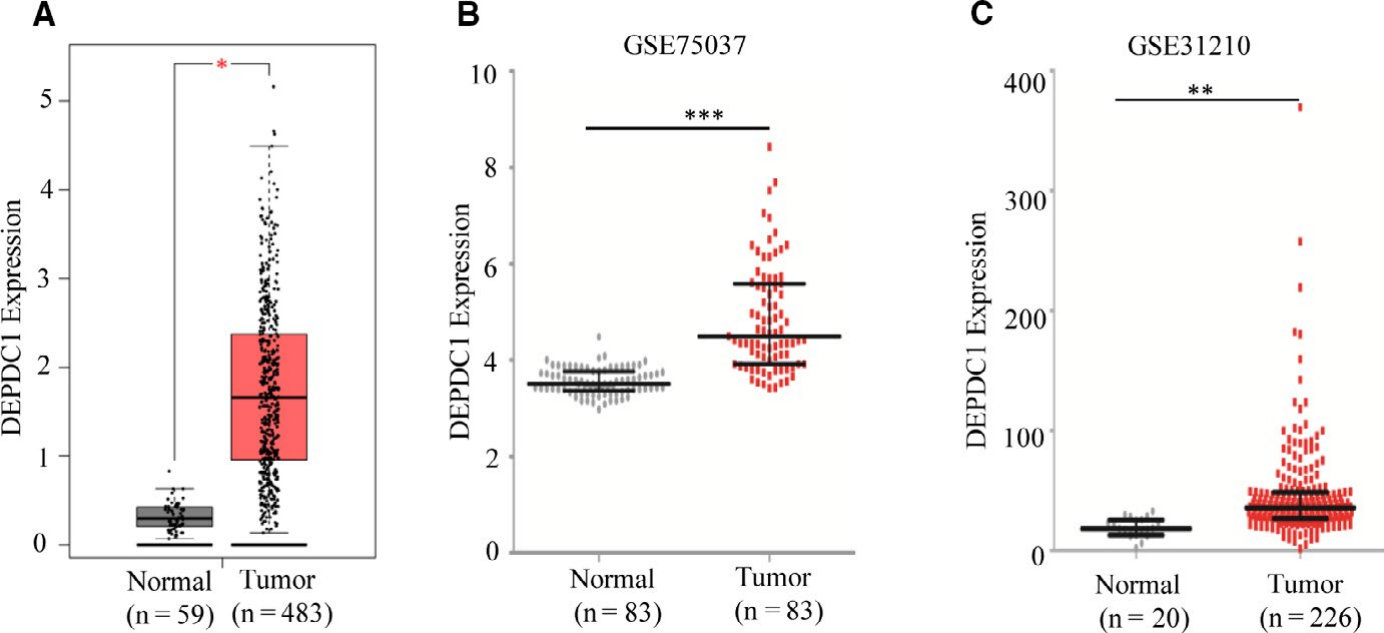
DEPDC1 expression is significantly up‐regulated in LUAD tissues. A, DEPDC1 expression was increased significantly in TCGA LUAD tissues. DEPDC1 expression was analysed by GEPIA software (*P < 0.05). B and C, DEPDC1 expression was increased significantly in LUAD tissues of GSE75037 and GSE31210 datasets from GEO database. DEPDC1 expression data were downloaded from GEO and analysed using GraphPad Prism (**P < 0.01, ***P < 0.001)

Our previous study found that DEPDC1 is expressed in multiple NSCLC cell lines and promotes proliferation of A549 cells,^12^ but its expression in LUAD tissues is not reported yet. To examine DEPDC1 expression in LUAD tissues, we analyse its expression in TCGA LUAD dataset using online tool GEPIA (gepia.cancer‐pku.cn).^19^ The results showed that DEPDC1 expression in LUAD tissues is significantly increased (*P* < 0.05) (Figure 1A). To confirm the results, we further analysed DEPDC1 expression in LUAD of two datasets GSE75037 and GSE 31210 from Gene Expression Omnibus (GEO) database. The results showed that DEPDC1 expression in LUAD tissues of these two datasets is significantly up‐regulated (P < 0.01 or *P* < 0.001 respectively, Figure 1B,C), which was consistent with TCGA LUAD results.

### 3.2 DEPDC1 high expression is correlated with unfavourable prognosis of LUAD patients

**FIGURE 2:**
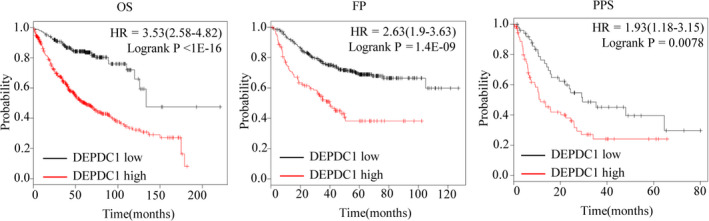
DEPDC1 high expression is correlated with unfavourable prognosis of LUAD patients. Overall survival (OS), first progression (FP) and post‐progression survival (PPS) of LUAD patients (N = 720) were analysed using online software Kaplan‐Meier Plotter (http://kmplot.com/analysis/) with auto selected best cut‐off. The results showed that the OS, FP and PPS of DEPDC1 high expression group were significantly decreased (OS: logrank *P* < 1e‐16, HR = 3.53(2.58‐4.82); FP: logrank *P* = 1.4e‐09, HR = 2.63 (1.9‐3.63); PPS: logrank *P* = 0.0078, HR = 1.93 (1.18‐3.15))

We used Kaplan‐Meier Plotter (kmplot.com) to analyse the correlation between the tumour DEPDC1 expression levels and the overall survival (OS), first progression (FP) and post‐progression survival (PPS) of LUAD patients (N = 720) in this database with auto select best cut‐off.^20^ The results showed that the OS, FP and PPS of DEPDC1 high expression group were significantly decreased than DEPDC1 low expression group (OS: logrank *P* < 1e‐16, HR = 3.53(2.58‐4.82); FP: logrank *P* = 1.4e‐09, HR = 2.63 (1.9‐3.63); PPS: logrank *P* = 0.0078, HR = 1.93 (1.18‐3.15)) (Figure 2), suggesting that DEPDC1 high expression is correlated with poor prognosis of LUAD patients.

### 3.3 DEPDC1 knockdown impairs proliferation, migration and invasion of A549 cells

**FIGURE 3:**
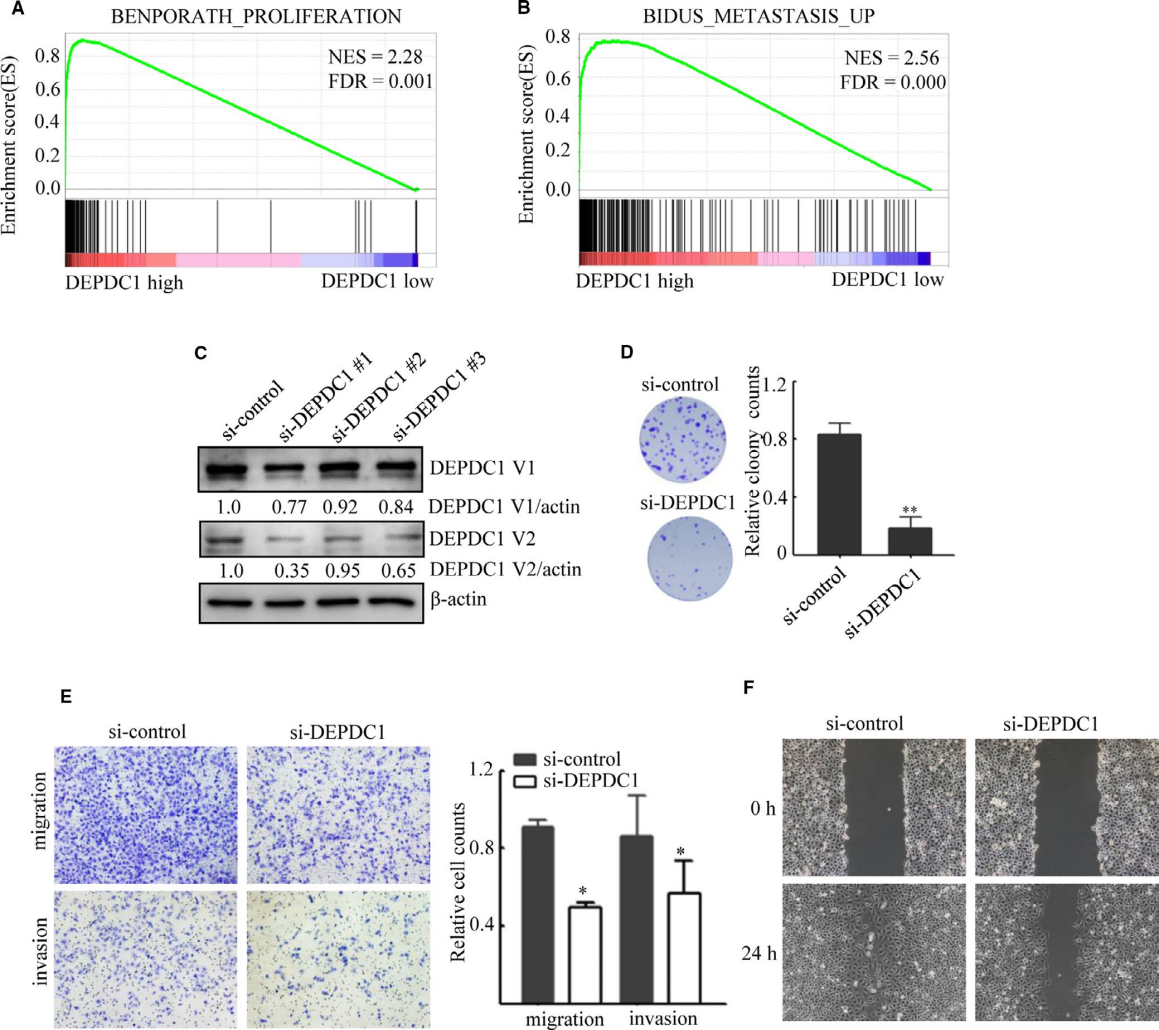
DEPDC1 knockdown inhibits proliferation, migration and invasion of A549 cells. A, The gene signatures of cell proliferation were highly enriched with DEPDC1 high expression in TCGA LUAD dataset. FDR, false discovery rate; NES, normalized enrichment score. B, The gene signatures of metastasis were highly enriched with DEPDC1 high expression in TCGA LUAD dataset. FDR, false discovery rate; NES, normalized enrichment score. C, The Western blot results demonstrated that DEPDC1 expression was suppressed by si‐DEPDC1, and si‐DEPDC1#1 had the strongest inhibitory effect. Bands were quantified using ImageJ and normalized to the loading control. D, DEPDC1 knockdown impairs the ability of proliferation. A549 cells were transfected with si‐DEPDC1 or control for 48 h, and then, colony formation assay was performed. **P < 0.01. E, DEPDC1 knockdown impairs the ability of invasion and migration. A549 cells were transfected with si‐DEPDC1 or si‐control for 24 h, and then, transwell (with or without Matrigel) assay was performed. *P < 0.05. F, DEPDC1 knockdown impairs the ability of migration. A549 cells were transfected with si‐DEPDC1 or si‐control for 24 h, and then, cell wound healing assay was conducted. The cells were photographed at 0 and 24 h, respectively

Our previous study showed that DEPDC1 is expressed in A549 cells and inhibits apoptosis,^12^ which might provide partial explanation to the correlation between DEPDC1 expression and poor prognosis. However, whether DEPDC1 affects other cellular phenotypes of LUAD cells remains unknown. To address this question, we analysed TCGA LUAD dataset using GSEA software to find the potential connection between DEPDC1 expression and cellular phenotypes. The bioinformatical analysis results demonstrated that the gene signatures of cell proliferation and metastasis were highly enriched with DEPDC1 high expression (Figure 3A,B).

We then performed in vitro experiments to study DEPDC1's function. Small interfering RNA (siRNA) was used to knock down DEPDC1 expression. Three siRNAs targeted DEPDC1 were designed and tested in A549 cells by detecting the DEPDC1 protein levels 48 hours after transfection. The results showed that DEPDC1 expression was dramatically reduced by si‐DEPDC1#1 compared with the control (Figure 3C). So si‐DEPDC1#1 was selected for further experiments. The colony formation assay, wound healing assay and Transwell assay were then conducted to determine the effects of DEPDC1 knockdown in A549 cells. The results showed that, compared with control group, DEPDC1 knockdown significantly reduced colony formation ability of A549 cells (Figure 3D), impaired the migration and invasion ability of A549 cells (Figure 3E,F). These findings confirmed bioinformatics results, suggesting that DEPDC1 promotes the proliferation, migration and invasion of A549 cells.

### 3.4 DEPDC1 knockdown induces autophagy by suppressing mTORC1 activity in LUAD cells

**FIGURE 4:**
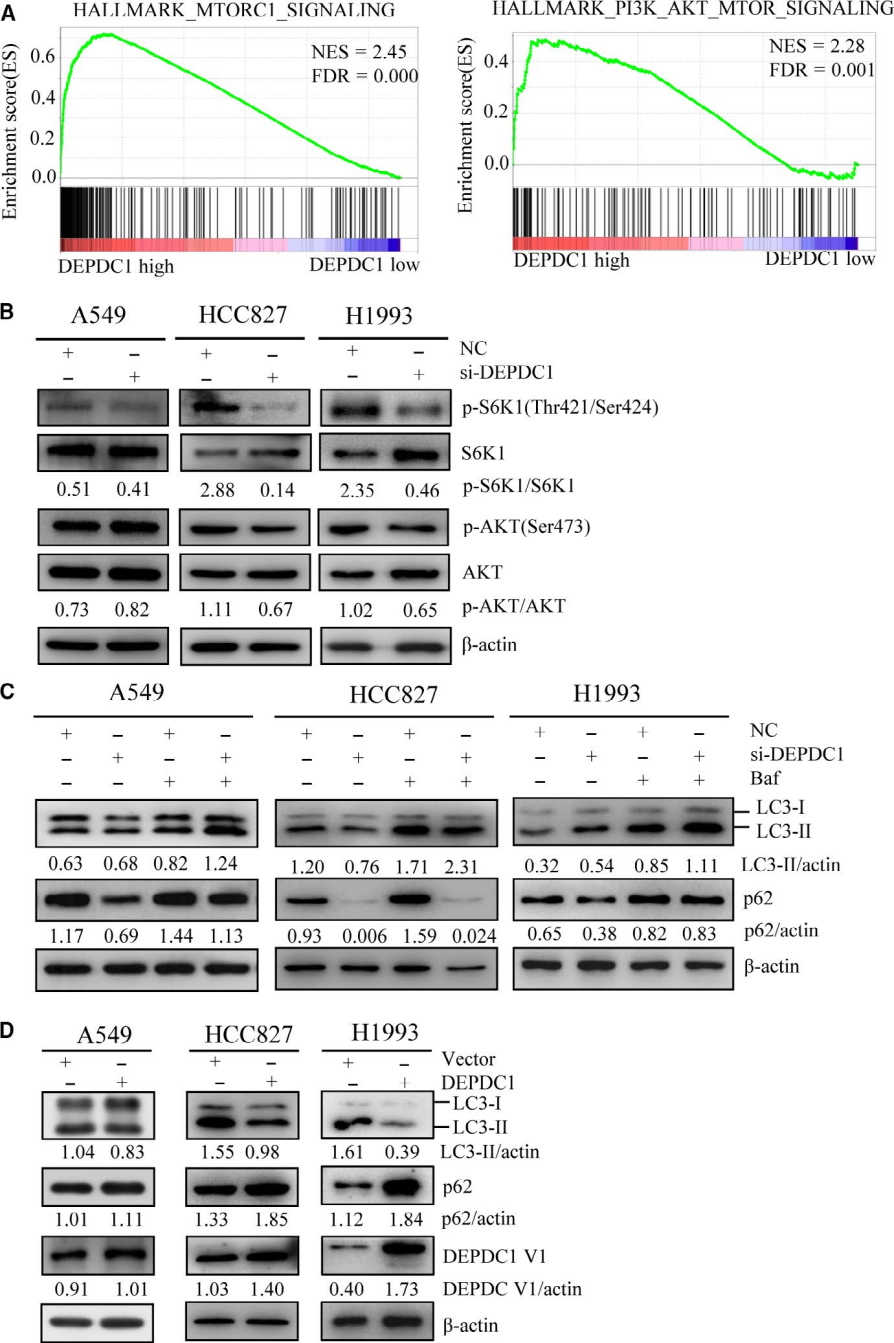
DEPDC1 knockdown induces autophagy by suppressing mTORC1 activity in LUAD cells. A, The gene sets of mTORC1 signalling pathway and PI3K/AKT/mTOR signalling pathway were highly enriched with DEPDC1 high expression. FDR, false discovery rate; NES, normalized enrichment score. B, DEPDC1 knockdown inhibited the phosphorylation of p‐S6K1 (Thr421/Ser424) in A549, HCC827 and H1993 cells, and suppressed p‐AKT (Ser473) in HCC827 and H1993 cells, but not in A549 cells. The cells were transfected with si‐DEPDC1 or control for 48 h, and the protein levels were detected by Western blot assay using the indicated antibodies. The bands were quantified using ImageJ and normalized to the loading control, and then, the ratio of phosphorylated protein and total protein was calculated. C, Autophagy was induced by DEPDC1 knockdown. The cells were transfected with si‐DEPDC1 or control for 48h, and then were treated with bafilomycin A1 or DMSO for 1 h prior to cell collection. The autophagic markers p62/SQSTM1 and LC3B were detected by Western blot assay. The results indicated enhanced autophagy upon DEPDC1 knockdown. The bands were quantified using ImageJ and normalized to the loading control. D, Autophagy was inhibited by DEPDC1 overexpression. The cells were transfected with DEPDC1 expression plasmids or empty vectors for 48 h. The autophagic markers p62/SQSTM1 and LC3B were detected by Western blot assay. The results showed that DEPDC1 overexpression inhibited the conversion of LC3B‐I to LC3B‐II and degradation of p62/SQSTM1. The bands were quantified using ImageJ and normalized to the loading control

As DEPDC1 acts as transcription repressor, we have been suggested there might be unidentified downstream effectors, which play pivotal role in cell survival and whose expression could be regulated by DEPDC1. To test this hypothesis and discover the potential pathways affected by DEPDC1, we performed gene set enrichment analysis of TCGA LUAD dataset using GSEA software by selecting hallmark gene sets. The results showed the gene sets of mTORC1 signalling pathway and PI3K/AKT/mTOR signalling pathway were highly enriched with DEPDC1 high expression (Figure 4A). To confirm the bioinformatical findings, we conducted in vitro experiment to test the effects of DEPDC1 expression change on the activities of mTORC1 and AKT. A549, HCC827 and H1993 cells were transfected with si‐DEPDC1 or si‐control, and 48 hours later, mTORC1 activity was analysed by detecting phosphorylation (Thr421/Ser424) of ribosomal protein S6 kinase beta‐1 (S6K1), a well‐known substrate of mTORC1, and AKT activity was accessed by determining its phosphorylation (Ser473). The results demonstrated that DEPDC1 knockdown inhibited phosphorylation of S6K1(Thr421/Ser424) in the three tested cell lines, and suppressed phosphorylation (Ser473) of AKT in HCC827 and H1993 cells, but did not have obvious effect on AKT phosphorylation (Ser473) in A549 cells (Figure 4B). Since mTORC1 is a key upstream inhibitor of autophagy, we then examined whether autophagy was affected upon DEPDC1 knockdown. The autophagic markers microtubule‐associated protein 1 light chain 3B (LC3B) and p62/sequestosome 1 (p62/SQSTM1) were detected by Western blot assay. The results showed that, in A549 and H1993, DEPDC1 knockdown resulted in the increase of LC3‐II and the decrease of p62/SQSTM1 protein compared with control siRNA group (Figure 4C). Moreover, lysosome inhibitor bafilomycin A1, which blocks autophagic flux, caused more apparent accumulation of LC3B‐II and p62/SQSTM1 in si‐DEPDC1 group than in control siRNA group. (Figure 4C). These results suggested that autophagy was enhanced upon DEPDC1 knockdown in these cells. However, in HCC827 cells, less LC3‐II and almost no p62/SQSTM1 were detected upon DEPDC1 knockdown, and bafilomycin A1 treatment caused more accumulation of LC3‐II but almost no accumulation of p62/SQSTM1 in si‐DEPDC1 group (Figure 4C). These findings indicated DEPDC1 knockdown caused more robust autophagy in HCC827 cells than in A549 and H1993 cells. The explanation for the conclusion is: the decrease of LC3‐II itself does not necessarily mean autophagy inhibition, because inhibited LC3‐I to ‐II conversion and enhanced LC3‐II degradation both may lead to the decrease of LC3‐II. Although less LC3‐II was observed in si‐DEPDC1 group in HCC827 cells, but bafilomycin A1 caused dramatic accumulation of LC3‐II, which indicated that the decrease of LC3‐II here was due to enhanced degradation. If LC3‐II decrease was due to autophagy inhibition, bafilomycin A1 treatment could not cause such dramatic accumulation of LC3‐II. Conversely, DEPDC1 overexpression suppressed p62/SQSTM1 degradation and the conversion of LC3B‐I to LC3B‐II in the tested three cells (Figure 4D), implying reduced autophagy. Taken together, these data suggested that DEPDC1 suppresses autophagy through regulating mTORC1 activity.

### 3.5 DEPDC1 up‐regulates RAS expression and enhances phosphorylation of ERK1/2 in LUAD cells

**FIGURE 5:**
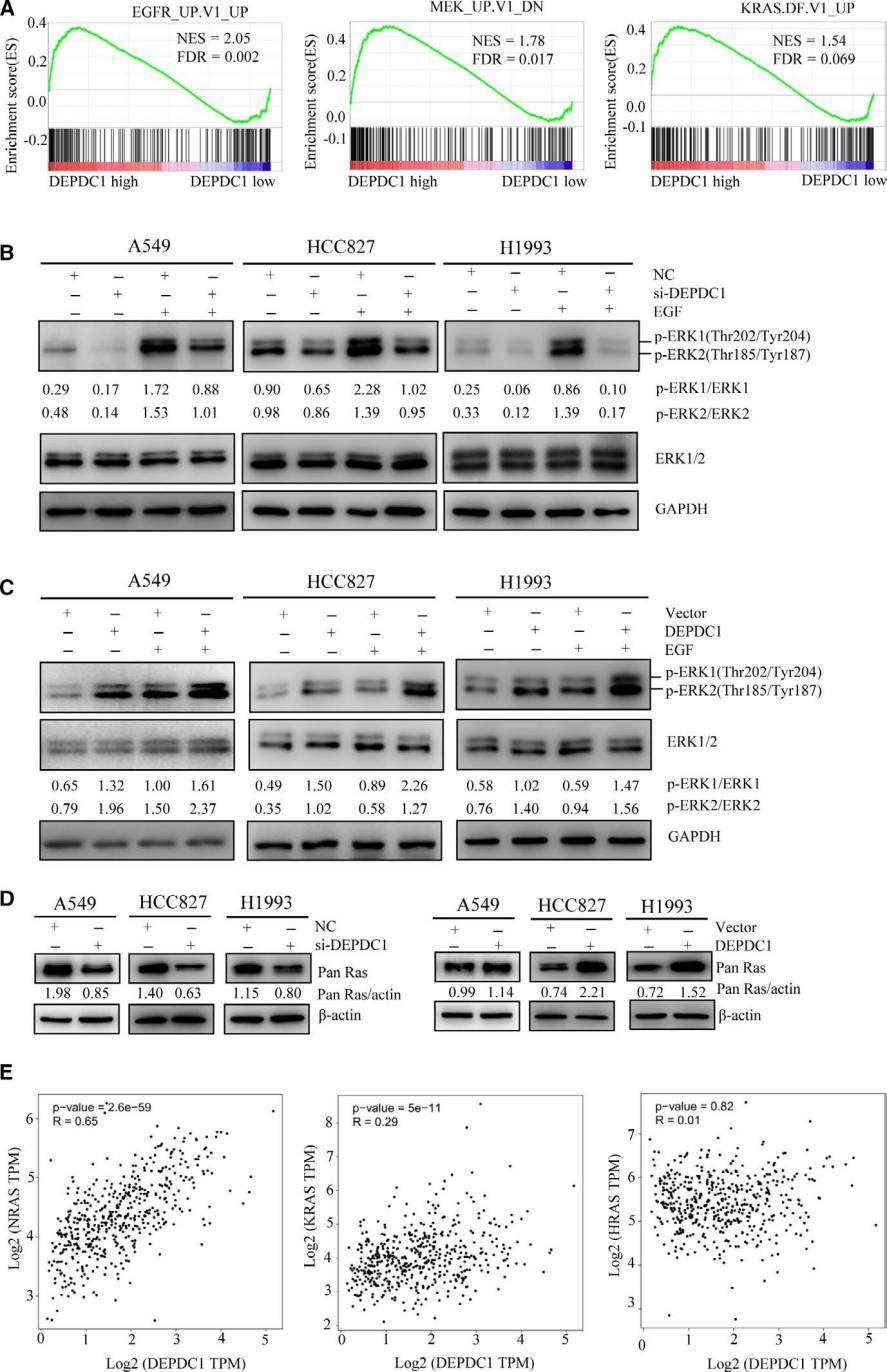
DEPDC1 promotes ERK1/2 phosphorylation and RAS expression in LUAD cells. A, The gene sets of EGFR, MEK and KRAS were highly enriched with DEPDC1 high expression. FDR, false discovery rate; NES, normalized enrichment score. B, DEPDC1 knockdown inhibits basal and EGF‐induced ERK phosphorylation (ERK1, Thr201/Tyr204; ERK2, Thr185/Tyr187). The cells were transfected with si‐DEPDC1 or control and cultured in media with 10% FBS for 36 h, followed with serum starvation 12 h and then EGF treatment (100 ng/mL) for 30 min. The phosphorylated and total ERK were detected by Western blot assay. The bands were quantified using ImageJ and normalized to the loading control, and then, the ratio of phosphorylated protein and total protein was calculated. C, DEPDC1 overexpression promotes basal and EGF‐induced ERK phosphorylation (ERK1, Thr201/Tyr204; ERK2, Thr185/Tyr187). The cells were transfected with DEPDC1 plasmids or empty vector and cultured in media with 10% FBS for 36 h, followed with serum starvation 12 h and then EGF treatment (100 ng/mL) for 30 min. The phosphorylated and total ERK were detected by Western blot assay. The bands were quantified using ImageJ and normalized to the loading control, and then, the ratio of phosphorylated protein and total protein was calculated. D, DEPDC1 knockdown suppressed RAS expression, and DEPDC1 overexpression promoted RAS expression. The cells were transfected with si‐DEPDC1 or DEPDC1 plasmids for 48 h, and the RAS protein was detected by Western blot assay. The bands were quantified using ImageJ and normalized to the loading control. E, The expression of NRAS and KRAS, but not HRAS, was correlated with DEPDC1 expression in TCGA LUAD tissues

Although AKT is a key upstream regulator of mTORC1,^21^ we observed that DEPDC1 knockdown induced autophagy but did not affect AKT activity in A549 cells, which indicated that there should be another pathway through which DEPDC1 regulates mTORC1 activity. To elucidate how mTORC1 activity is regulated by DEPDC1 without AKT involvement, we analysed TCGA LUAD dataset again using GSEA software to discover oncogenic gene sets that might be involved in DEPDC1 regulating mTORC1. The bioinformatical analysis results demonstrated that the gene sets of EGFR, MEK and KRAS were highly enriched with DEPDC1 high expression (Figure 5A). Since ERK1/2 is a key common component downstream of these three signalling pathways, we conducted experiments to detect ERK1/2 phosphorylation change under the conditions of DEPDC1 knockdown and overexpression. A549, HCC827 and H1993 cells were transfected with si‐DEPDC1 or si‐control for DEPDC1 knockdown, or with DEPDC1 plasmids or empty vector plasmids for DEPDC1 overexpression. Then, the cells were cultured in media with 10% FBS for 36 hours, followed by serum starvation for 12 hours and then 30 minutes EGF (100 ng/mL) treatment. Finally, the cells were harvested and ERK1/2 phosphorylation was determined by Western blot assay. The results showed that DEPDC1 knockdown inhibited basal and EGF‐induced ERK1/2 phosphorylation compared with the control group (Figure 5B). In contrast, DEPDC1 overexpression promoted basal and EGF‐induced ERK1/2 phosphorylation compared with the control group (Figure 5C). These data indicate that DEPDC1 can stimulate ERK1/2 phosphorylation through the EGFR/ERK1/2 signalling pathway. RAS protein was also detected under the conditions of DEPDC1 knockdown and overexpression, and the results showed that RAS expression was suppressed by DEPDC1 knockdown, but enhanced by DEPDC1 overexpression (Figure 5D), suggesting DEPDC1 can up‐regulate RAS expression. To further verify this finding, the correlation between DEPDC1 expression and RAS expression in LUAD tissues was analysed by GEPIA, and the data showed that NRAS and KRAS, but not HRAS, were positively correlated with DEPDC1 expression in LUAD tissues (Figure 5E). Taken together, these findings suggest that DEPDC1 can up‐regulate RAS expression and therefore enhance ERK1/2 activity.

### 3.6 Inhibition of RAS‐ERK1/2 signalling stimulates autophagy in LUAD cells

**FIGURE 6:**
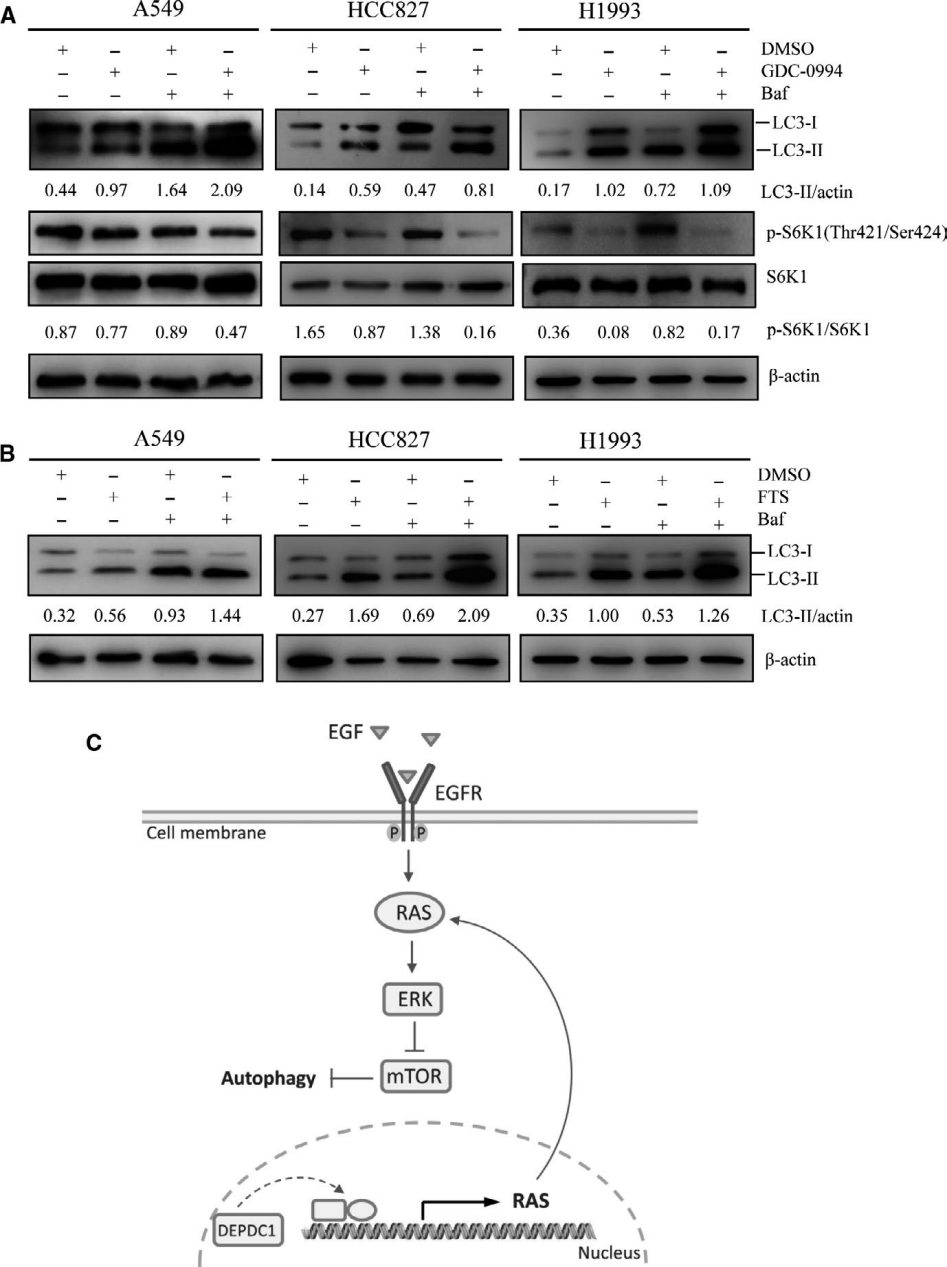
Inhibition of RAS‐ERK1/2 signalling stimulates autophagy in LUAD cells. A, ERK1/2 inhibitor GDC‐0994 (25 µmol/L) induced autophagy in LUAD cells. The cells were treated with GDC‐0994 (25 µmol/L) for 24 h, followed by bafilomycin A1 treatment for 1 h. The autophagic marker LC3B as well as p‐S6K1 (Thr421/Ser424) and total S6K1 were detected by Western blot assay. The bands were quantified using ImageJ and normalized to the loading control, and then, the ratio of phosphorylated protein and total protein was calculated. B, Inhibition of RAS enhanced autophagy. The cells were treated with RAS inhibitor FTS (150 µmol/L) for 24 h, followed by bafilomycin A1 treatment for 1 h. The autophagic marker LC3B was detected by Western blot assay. The bands were quantified using ImageJ and normalized to the loading control. C, Schematic diagram of the proposed mechanism by which autophagy is inhibited by DEPDC1 in LUAD cells

We observed DEPDC1 knockdown enhanced autophagy, and DEPDC1 knockdown lowered RAS expression and ERK1/2 activity in the aforementioned experiments. But it is unclear whether DEPDC1 regulates autophagy through RAS‐ERK1/2 signalling yet. To answer this question, A549, HCC827 and H1993 cells were treated with ERK1/2 inhibitor GDC‐0994 (25 µmol/L) or RAS inhibitor farnesylthiosalicylic acid (FTS, 150 µmol/L) for 24 hours, followed by autophagy inhibitor bafilomycin A1 treatment for 1 hour. Finally cells were harvested and Western blot assay was employed to determine the protein levels. In ERK1/2 inhibitor experiments, the autophagic markers LC3B, and p‐S6K1 (Thr421/Ser424) and total S6K1 were detected. The results showed that, compared with the control group, inhibition of ERK1/2 led to the increase of LC3B‐II, and bafilomycin A1 treatment resulted in robust accumulation of LC3B‐II. Meanwhile, decreased p‐S6K1 (Thr421/Ser424) was observed upon inhibition of ERK1/2 (Figure 6A). These data suggest that inhibition of ERK1/2 enhance autophagy, which indicates that ERK1/2 can inhibit autophagy through mTORC1 in the tested cells. In RAS inhibitor experiments, the autophagic marker LC3B was examined and the results showed that, compared with the control experiments, inhibition of RAS enhanced the conversion of LC3B‐I to LC3B‐II, and bafilomycin A1 treatment resulted in remarkable accumulation of LC3B‐II (Figure 6B), implying that inhibition of RAS can enhance autophagy. Together, these findings suggest that inhibition of RAS‐ERK1/2 signalling stimulates autophagy in LUAD cells (Figure 6C).

## 4 DISCUSSION

The present study revealed that DEPDC1 plays a pivotal role in tumorigenesis of LUAD by analysing DEPDC1 expression in three LUAD datasets from public databases and the underlying mechanisms. Our data demonstrated that DEPDC1 expression is significantly up‐regulated in LUAD, and its high expression is correlated with unfavourable prognosis. These findings are consistent to the previous findings regarding DEPDC1's role in other types of tumours.^7^, ^8^, ^9^ Since increased cellular proliferation, migration and invasion are hallmarks of cancer cells,^22^ our observation that DEPDC1 knockdown impairs proliferation, migration and invasion of A549 cells provides cellular evidence to address how DEPDC1 negatively affects LUAD patients' prognosis.

The present study uncovered a novel mechanism that DEPDC1 could up‐regulate the expression of RAS, which ultimately inhibits autophagy through RAS‐ERK‐mTORC1 pathway (Figure 6C). To our knowledge, this is the first study found DEPDC1 promotes RAS expression, and thus serves as an autophagy inhibitor. Although autophagy plays a dual role in tumorigenesis,^18^ based on a previous report that autophagy inhibition was associated with increased clonogenic survival in NSCLC cells both in vitro and in vivo,^23^ it is more likely that DEPDC1 inhibition of autophagy should have positive contribution to LUAD tumorigenesis. Two recent studies demonstrated that, in pancreatic cancer, combinations of pharmacologic inhibitors that concurrently block both ERK and autophagy may be effective treatments for pancreatic cancer,^24^, ^25^ so it is worth to study in the future whether concurrent blockade of both ERK and autophagy will have promising effects on LUAD with DEPDC1 high expression. RAS proteins (including KRAS, NRAS and HRAS), together with their downstream signalling proteins, play crucial roles in the control of normal cellular homeostasis.^26^ RAS signalling is commonly dysregulated in tumours due to genetic alteration of RAS gene or other no‐genetic events.^26^ RAF‐MEK‐ERK and PI3K‐AKT‐mTOR are the canonical signalling pathways, which have well‐validated contributions to RAS pro‐proliferation, pro‐survival and pro‐metastasis functions in cancer.^27^ Therefore, our findings provide novel molecular evidence how DEPDC1 promotes proliferation, migration and invasion of LUAD cells. As RAS may activate PI3K, our findings might lend evidence to explain how PI3K/AKT/mTOR signalling is hyper‐activated by DEPDC1 in breast cancer cells, which was reported in a previous study.^10^ Surprisingly, there was no obvious alteration of AKT phosphorylation upon DEPDC1 knockdown in A549 cells in our experiments, which might be due to specific cellular context of this cell line. Since RAS can stimulates FOXM1 expression,^28^ our findings also might be used to explain how FOXM1 is up‐regulated by DEPDC1 in triple negative breast cancer cells, which was observed in another study.^28^ Moreover, our study indicates the inhibitors of RAS pathway might be potential therapeutic agents for LUAD with DEPDC1 overexpression.

In summary, the current study revealed DEPDC1 is highly expressed in LUAD tissues, and its high expression is associated with unfavourable clinical outcomes of LUAD patients. This study discovered DEPDC1 may promote RAS expression to inhibit autophagy, which casts light on understanding the underlying mechanisms of DEPDC1. However, in order to completely elucidate the role of DEPDC1 in tumorigenesis, it is necessary to investigate how RAS expression is regulated by DEPDC1 in the future.

## CONFLICT OF INTEREST

The authors declare that there is no conflict of interest.

## AUTHOR CONTRIBUTIONS

**Wei Wang:** Data curation (equal); formal analysis (equal); investigation (equal); methodology (equal); validation (equal); visualization (equal); writing – original draft (equal). **Aili Li:** Formal analysis (equal); investigation (equal); methodology (equal); validation (equal). **Xiaodan Han:** Investigation (equal); methodology (equal); validation (equal). **Qingqing Wang:** Formal analysis (equal); investigation (equal); methodology (equal); validation (equal); visualization (equal). **Jinyong Guo:** Investigation (equal); validation (equal). **Youru Wu:** Investigation (equal); methodology (equal); validation (equal). **Chen Wang:** Investigation (equal); methodology (equal). **Guojin Huang:** Conceptualization (lead); formal analysis (lead); funding acquisition (lead); investigation (equal); methodology (equal); software (equal); supervision (lead); visualization (lead); writing – review and editing (lead).

## Data Availability

The data that support the findings of this study are available from the corresponding author upon reasonable request.
